# Long‐term efficacy and safety of programmed death‐1 (PD‐1) antibody alone in relapsed/refractory human immunodeficiency virus‐associated Hodgkin lymphoma

**DOI:** 10.1002/jha2.448

**Published:** 2022-05-04

**Authors:** Chaoyu Wang, Fanlin Zhou, Lingqian Zhang, Tingting Liu, Yingyu Nan, Yao Liu

**Affiliations:** ^1^ Department of Hematology Oncology Chongqing University Cancer Hospital, Chongqing Key Laboratory of Translational Research for Cancer Metastasis and Individualized Treatment Chongqing China; ^2^ Department of Pathology Chongqing University Cancer Hospital, Chongqing Key Laboratory of Translational Research for Cancer Metastasis and Individualized Treatment Chongqing China

**Keywords:** HIV, Hodgkins lymphom, PD‐1

## Abstract

We report a young patient initially diagnosed with human immunodeficiency virus (HIV)‐associated Hodgkin lymphoma (HL), and received six cycles of ABVD chemotherapy regimens and involvement field irradiation therapy. However, the disease progressed after two months later, and then received second line GDP regimen. Unfortunately, after five cycles of GDP, the patient progression disease (PD) again. The patient was then offered sintilimab alone. After 8 cycles, the patient received complete response (CR) and no 3/4 grade toxicity. Currently, at a follow‐up period of four years, he is still alive with CR and no lymphoma‐related symptoms. This case demonstrates the feasibility of sintilimab antibody in relapsed/refractory HIV‐associated Hodgkin lymphoma.

1

We have experienced a case of relapsed/refractory (R/R) human immunodeficiency virus (HIV)‐associated Hodgkin lymphoma (HL), in which achieved complete response (CR) and has no lymphoma‐related symptoms more than 3 years after sintilimab treatment. To the best of our knowledge, this is the first report of R/R HIV‐associated HL patient on sintilimab.

A 26‐year‐old man was diagnosed with HIV in March 2015. After initiation use of combination antiretroviral therapy (cART) with elvitegravir, cobicistat, emtricitabine, and tenofovir disoproxil, he maintained good disease control for the next 3 years, with consistently undetectable viral loads and no serious infections. In April 2018, the patient presented with a 1‐month history of enlarged lymph nodes in the both side cervical regions without pain and fever. Physical examination showed soft tissue mass in the bilateral cervical, axilla, and inguinal region. His CD4 count was 209 cells/mm^3^ and serum lactate dehydrogenase was elevated (617 U/L), while other laboratory data, including full blood count, liver function tests, renal function and viral loads, were normal. ^18^F‐fluorodeoxyglucose (FDG) positron emission tomography (PET)/computed tomography (CT) scanning showed increased FDG avidity involving the mediastinum, right lung hilum, bilateral cervical, axilla, inguinal region, and spleen. PET‐CT also showed a FDG‐avid abdominal mass, suggesting lymphadenopathy (Figure 1A). A CT‐guided biopsy of the left cervical lymph nodes was performed and showed classical HL (cHL) (lymphocyte‐rich type). Lymphoma cells were positive for PAX‐5, CD15, CD30, MUM‐1, Epstein–Barr virus encoded RNA (EBER), and negative for CD3, CD5, CD7, CD20, CD21. Ki67 was expressed by more than 80% of lymphoma cells. Bone marrow aspiration showed no infiltration. A diagnosis of stage IIISB HIV‐associated cHL was made, and the patient received six cycles of ABVD (doxorubicin, bleomycin, vinblastine, and dacarbazine) and treated with 46 Gy (23 fractions) of involvement field irradiation to the right inguinal lesion.

**FIGURE 1:**
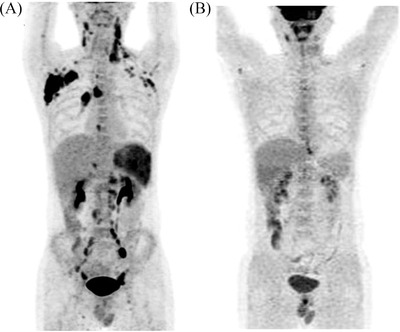
Comparison of curative effect of patients before (A) and after (B) progression disease programmed death‐1 (PD‐1) antibody alone treatment through positron emission tomography (PET)/computed tomography (CT) image examination

Two months later, the mass in the right lung hilum had increased, PET/CT scanning showed increased FDG avidity, and Deauville scored 5. The patient refused autologous hematopoetic stem cell transplant (auto‐HCT) and received second line GDP (gemcitabine, dexamethasone, and cisplatin) regimen chemotherapy. Unfortunately, after five cycles of GDP, PET/CT scanning showed new emerging enlargement lymph node in the retroperitoneum with Deauville scored 5, and progression disease (PD) again. The patient was then offered programmed death‐1 (PD‐1) antibody alone (sintilimab). After eight cycles (200 mg, every 2 weeks), the posttreatment PET/CT (Figure 1B) demonstrated resolution of previous abnormal uptake and nodal size consistent with CR and no 3/4 grade toxicity. The viral loads consistently undetectable during therapy. And, the immune system function, including CD4 count, CD8 count, NK cell count and B lymphocyte, was normal during the cART. Currently, at a follow‐up period of 4 years, the patient is alive, has still no lymphoma‐related symptoms, and has received a total of 32 cycles of sintilimab by the time of this report.

HIV‐associated cancers are divided into acquired immune deficiency syndrome (AIDS)‐defining and non‐AIDS‐defining malignancies based on the coincidence rate among HIV‐infected patients [1]. The HL is one of the most common non‐AIDS‐defining malignancies. Powles demonstrated that the incidence of HL in patients living with HIV was increased by 5–20 times compared with the HIV‐negative population [2]. Before cART, the outcomes of the HIV‐associated HL were poor. Now, with the wide use of cART and standard chemotherapy, the prognosis of these patients is similar to that of the general population [3]. However, the outcome of patients with HIV‐associated HL becomes very poor when the lymphoma relapsed or is refractory to the first‐line chemotherapy [4]. So far, there is no standard second‐line therapy.

AutoHCT has been established as the standard treatment for managing patients with chemotherapy‐sensitive, R/R HL [5]. Meanwhile, some clinical trials also demonstrated the feasibility of AutoHCT in HIV‐positive patients with R/R HL [6, 7]. So, relapses in HIV‐infected patients with HL can be treated with the same strategies as HIV‐negative patients. The patient refused auto‐HCT and again PD after received second line GDP regimen chemotherapy. The new immunotherapy, such as checkpoint inhibition with anti‐PD‐1 drugs, has been used in some patients and is currently under investigation in clinical trial. After treated with sintilimab, our patient achieved CR within 2 months. And he is still alive with CR and no lymphoma‐related symptoms more than 3 years. This case demonstrates the feasibility of programmed death‐1 (PD‐1) antibody in R/R HIV‐associated HL.

## CONFLICT OF INTEREST

The authors declare they have no conflicts of interest.

## AUTHOR CONTRIBUTIONS

Chaoyu Wang wrote the manuscript. Lingqian Zhang, Tingting Liu and Yingyu Nan provided patient care. Fanlin Zhou made the pathological diagnosis. Yao Liu was the chief of our department and revised the manuscript. All the authors reviewed the manuscript and provided final approval.

## ETHICS STATEMENT

The studies involving human participants were reviewed and approved by the institutional review board of Chongqing University Cancer Hospital. The patient provided his written informed consent to participate in this study.
